# Predictive value of volumetric parameters based on ^18^F-PSMA-1007 PET/CT for prostate cancer metastasis

**DOI:** 10.3389/fonc.2024.1335205

**Published:** 2024-02-26

**Authors:** Yanmei Li, Jian Chen, Xiaojuan Wang, Pengfei Yang, Jiqin Yang, Qian Zhao, Juan Li

**Affiliations:** ^1^ Department of Nuclear Medicine, General Hospital of Ningxia Medical University, Yinchuan, China; ^2^ College of Clinical Medicine, Ningxia Medical University, Yinchuan, China; ^3^ Department of Radiology, General Hospital of Ningxia Medical University, Yinchuan, China; ^4^ Department of Medical Instrumentation, General Hospital of Ningxia Medical University, Yinchuan, China

**Keywords:** ^18^F-PSMA-1007, metastasis, positron emission tomography, predictive value, prostate cancer, volumetric parameters

## Abstract

**Purpose of the report:**

To explore the value of ^18^F-labeled prostate-specific membrane antigen (PSMA-1007) positron emission tomography (PET)/computed tomography (CT), the maximum standardized uptake value (SUVmax) of the primary tumor, prostate PSMA-tumor volume (PSMA-TVp), and prostate total lesion PSMA (TL-PSMAp) for predicting prostate cancer (PCa) metastasis and follow-up evaluation in primary PCa lesions.

**Materials and methods:**

^18^F-PSMA-1007 PET/CT data of 110 consecutive newly diagnosed PCa patients were retrospectively analyzed. Patients were divided into non-metastatic, oligometastatic, and extensive metastatic groups. The predictive power was assessed using the receiver operating characteristic curve. Multi-group one-way analysis of variance and *post-hoc* tests were used to compare the groups. Patients were monitored post-therapy to evaluate treatment effectiveness.

**Results:**

Among the 110 patients, 66.4% (73) had metastasis (29 oligometastatic, 44 extensive metastasis). AUCs for Gleason score (GS), total prostate-specific antigen(TPSA), SUVmax, TL-PSMAp, and PSMA-TVp were 0.851, 0.916, 0.834, 0.938, and 0.923, respectively. GS, TPSA, SUVmax, TL-PSMAp, and PSMA-TVp were significantly different among the groups. In the *post-hoc* tests, differences in GS, TPSA, SUVmax, TL-PSMAp, and PSMA-TVp between the non-metastatic and oligometastatic groups and non-metastatic and extensive metastatic groups were significant (P<0.010). Differences in TL-PSMAp and PSMA-TVp between oligometastatic and extensive metastatic groups were significant (P=0.039 and 0.015, respectively), while those among GS, TPSA, and SUVmax were not. TL-PSMAp and PSMA-TVp distinguished between oligometastatic and extensive metastases, but GS, TPSA, and SUVmax did not. In individuals with oligometastasis, the implementation of active treatment for both primary and metastatic lesions may result in a more favorable prognosis.

**Conclusions:**

^18^F-PSMA-1007 PET/CT volumetric parameters PSMA-TVp and TL-PSMAp can predict PCa oligometastasis.

## Introduction

Metastasis is the main cause of complications and death in patients with prostate cancer (PCa) (1, 2). The 5-year survival rates for localized and metastatic PCa are 100% and <30%, respectively (3). More than 70% of PCa patients in China are diagnosed at the middle or advanced stages and 30% of patients have distant metastases at the time of first diagnosis, resulting in poor overall prognosis in these patients (4, 5). Improving the survival rate and quality of life in patients with metastatic PCa is a popular research topic.

Owing to the great diversity and heterogeneity among primary PCa tumors and different metastatic foci, there are obvious biological differences between localized and extensive metastases of PCa. Some scholars have proposed the concept of “oligometastatic PCa” (6, 7). The oligometastatic state was first proposed by Hellman and Weichselbaum in 1995 and then widely applied. This state refers to an intermediate stage between localized and widespread metastases (8). At this stage, the biological aggressiveness of the tumor is mild, with a limited number of metastatic tumors and limited metastatic organs (9), and has not yet spread throughout the body. At present, the definition of oligometastatic PCa has not been clearly established, particularly concerning the extent and distribution of metastatic lesions. However, a majority of studies have established a threshold of 3-5 metastatic sites on conventional imaging as the delineating criterion (10). Further studies have shown that the management of primary and metastatic lesions in individuals diagnosed with oligometastatic PCa tends to enhance both their quality of life and overall survival (11, 12).

In the past, the diagnosis of oligometastases of PCa was based on traditional imaging examinations such as whole-body bone scintigraphy and magnetic resonance imaging (MRI). However, these traditional diagnostic tools have great limitations in the accurate staging of PCa. According to previous reports, the sensitivity of bone scans for metastatic bone lesions is only 65%, and the sensitivity of computed tomography (CT) and conventional MRI for detecting lymph node metastases in PCa is approximately 36% (13, 14). Therefore, new imaging techniques are needed to identify oligometastatic states in patients with PCa.

Molecular imaging has greatly impacted the management of PCa patients, with PET/CT using 11C-choline or 18F-choline commonly used to detect metastases of PCa (15). The superior predictive value of PET/CT utilizing choline or acetate tracers, in comparison to CT and bone scans, establishes these tracers as promising diagnostic instruments for oligometastatic prostate cancer (16, 17). Due to possible uptake artifacts from inflammation and degenerative bone disease, they may not be as accurate in detecting nodal and bone metastases (18).

Prostate-specific membrane antigen (PSMA), a type II transmembrane protein with high expression in PCa cells and higher expression in cancer cells in advanced stages of cancer and anti-androgen therapy, is a novel biological PCa target (19, 20). PSMA-based positron emission tomography (PET)/CT imaging has shown great promise in PCa diagnosis, staging, prognosis, and recurrence monitoring (21); its most commonly used modality is ^68^Ga-PSMA-11. The maximum standardized uptake value (SUVmax), which is one of the most commonly applied parameter in ^68^Ga-PSMA-11 PET/CT, has been studied in depth and has been shown to be of great application value in the stage and risk stratification of PCa (22). Nevertheless, SUVmax fails to offer a comprehensive assessment of the entire tumor volume and does not accurately represent the extent of tumor burden; therefore, it has certain limitations in the prognosis assessment of PCa. Increasing evidence suggests that tumor invasiveness is related to tumor volume and burden, the tumor volume is considered as an independent risk factor in biochemical recurrence of PCa (23). Therefore, volume parameters such as prostate PSMA-tumor volume (PSMA-TVp) and prostate total lesion PSMA (TL-PSMAp) are also being studied to overcome the above-mentioned limitations (24, 25). However, there are few studies on the clinical application of tumor volume with PSMA PET/CT.

^18^F-PSMA-1007 is a recently developed radiopharmaceutical. Compared to other PSMA PET/CT tracers, it has the advantages of a long half-life, a high yield, and non-urinary excretion, making it more favorable for the evaluation of primary lesions and metastases of PC (26, 27). Therefore, this study aimed to analyze the correlation of the PSMA volumetric parameters (PSMA-TVp) and prostate total lesion PSMA (TL-PSMAp) in newly diagnosed PCa lesions using ^18^F-PSMA-1007 PET/CT imaging with clinical and pathological characteristics in comparison with SUVmax to determine the clinical value of volumetric parameters in the metastatic state of PCa. The objective is to provide a reference for selecting treatment methods and evaluating prognosis in patients with oligometastatic PCa.

## Materials and methods

### Patients

The data of 227 patients with PCa who underwent ^18^F-PSMA-1007 PET/CT between December 2019 and June 2023 were retrospectively analyzed. Patients who had complete data (including age, smoking history, previous history, family history of tumor, BMI, TPSA, MRI, whole body bone scintigraphy, pathology, etc.) and who underwent transrectal ultrasound guided prostate biopsy (TRUS) or radical prostatectomy to obtain pathological results were included. If the patient subsequently underwent radical prostatectomy, we compared the pathologic results with TRUS and selected a higher-grade Gleason score (GS) as the criterion for all PCa patients according to the European Association of Urology and the International Society of Urological Pathology 2014 classification criteria. Those with any of the following criteria were excluded: 1) PCa concomitant with other malignancies; 2) >1 month between total prostate-specific antigen (TPSA) test, prostate biopsy, and ^18^F-PSMA-1007 PET/CT examination; 3) treatment with endocrine first- or second-generation antiandrogen therapy (e.g. bicalut-amide) or surgical treatment (prostatectomy) or systemic therapy prior to imaging; and 4) no PSMA uptake in primary lesions of PCa.

**Figure 1:**
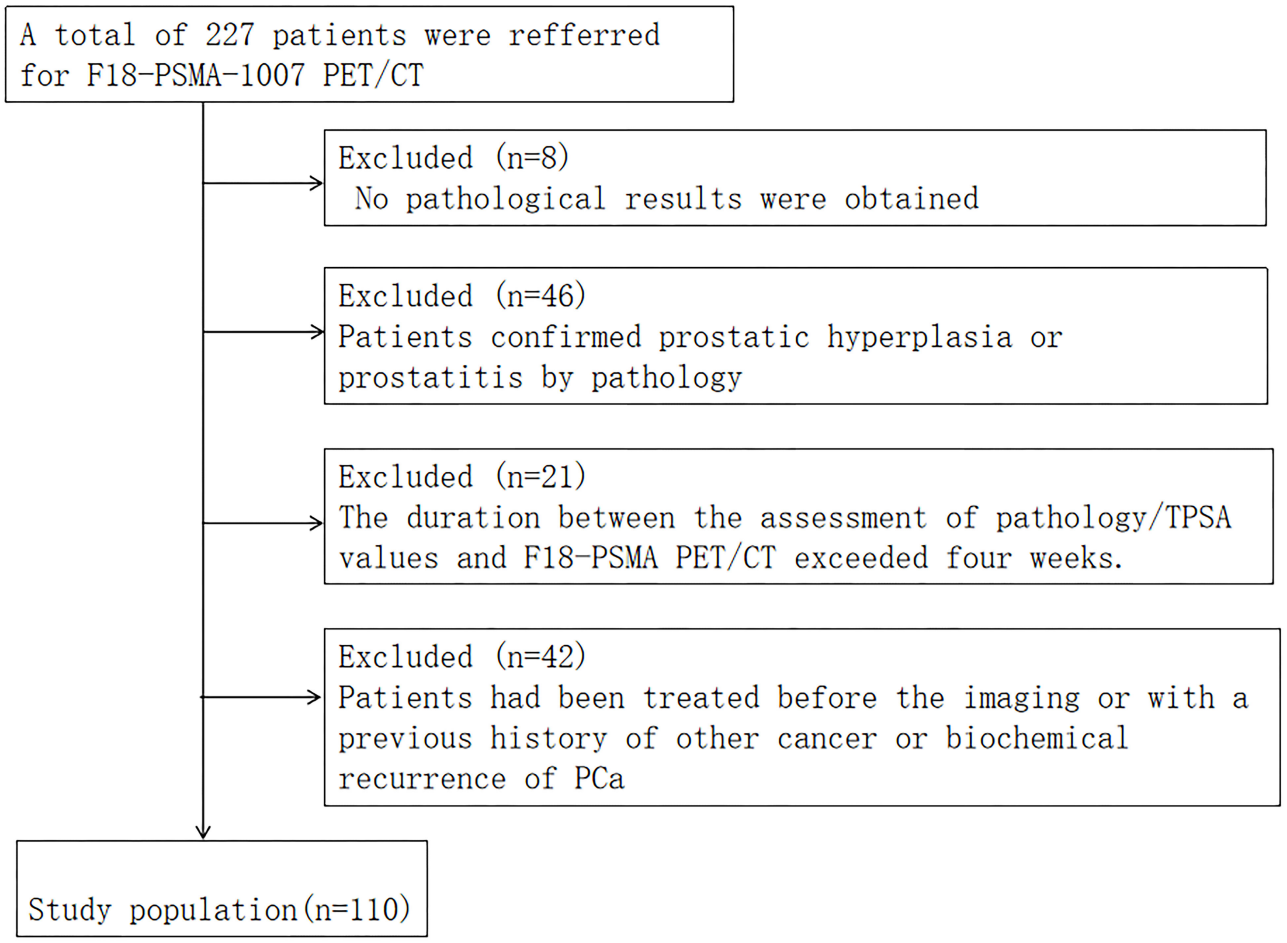
Flow chart of patient selection.

Finally, 110 patients were included (**Figure 1**). The study protocol was approved by the Hospital Ethics Committee (Ethics Approval No. 2020-083, 2020-876) and followed the principles outlined in the Declaration of Helsinki. Prior to participation, all volunteers provided their informed consent by signing appropriate documentation. Follow-up mainly included telephone calls and outpatient appointments.

### Methods of examination

All patient examinations were performed according to PCa PSMA PET/CT imaging Guidelines (EANM Guidelines/SNMMI Procedure Standard 2.0). The ^18^F-PSMA-1007 PET/CT examination was performed by an experienced nuclear medicine technician licensed to work with large equipment. The study employed a GE Discovery VCT PET-CT (64-row CT) scanner, and the equipment was qualified for quality control. The ^18^F-PSMA-1007 was synthesized using PET-IFB-X5 (Shaanxi Zhengze Biotechnology Co., Ltd.). The purity of ^18^F-PSMA-1007 was assessed using high-performance liquid chromatography, yielding a result of ≥95%.The injected ^18^F-PSMA-1007 was 4.0 MBq/kg. A whole-body scan was performed approximately 60–90 min after injection, followed by a spiral CT scan ranging from the cranial roof to the middle femur.

The scanning parameters included a tube voltage of 140 kV, tube current of 150 mA, layer thickness of 3.75 mm, pitch of 0.875, and matrix size of 512 × 512. PET scans were conducted using a three-dimensional mode, employing a matrix of 128 × 128, and allocating 2.5 minutes per bed. The total number of beds scanned ranged from 7 to 9. CT data were utilized to correct for attenuation in the PET images, followed by image reconstruction and fusion.

### ^18^F-PSMA-1007 PET/CT image analysis

**Figure 2:**
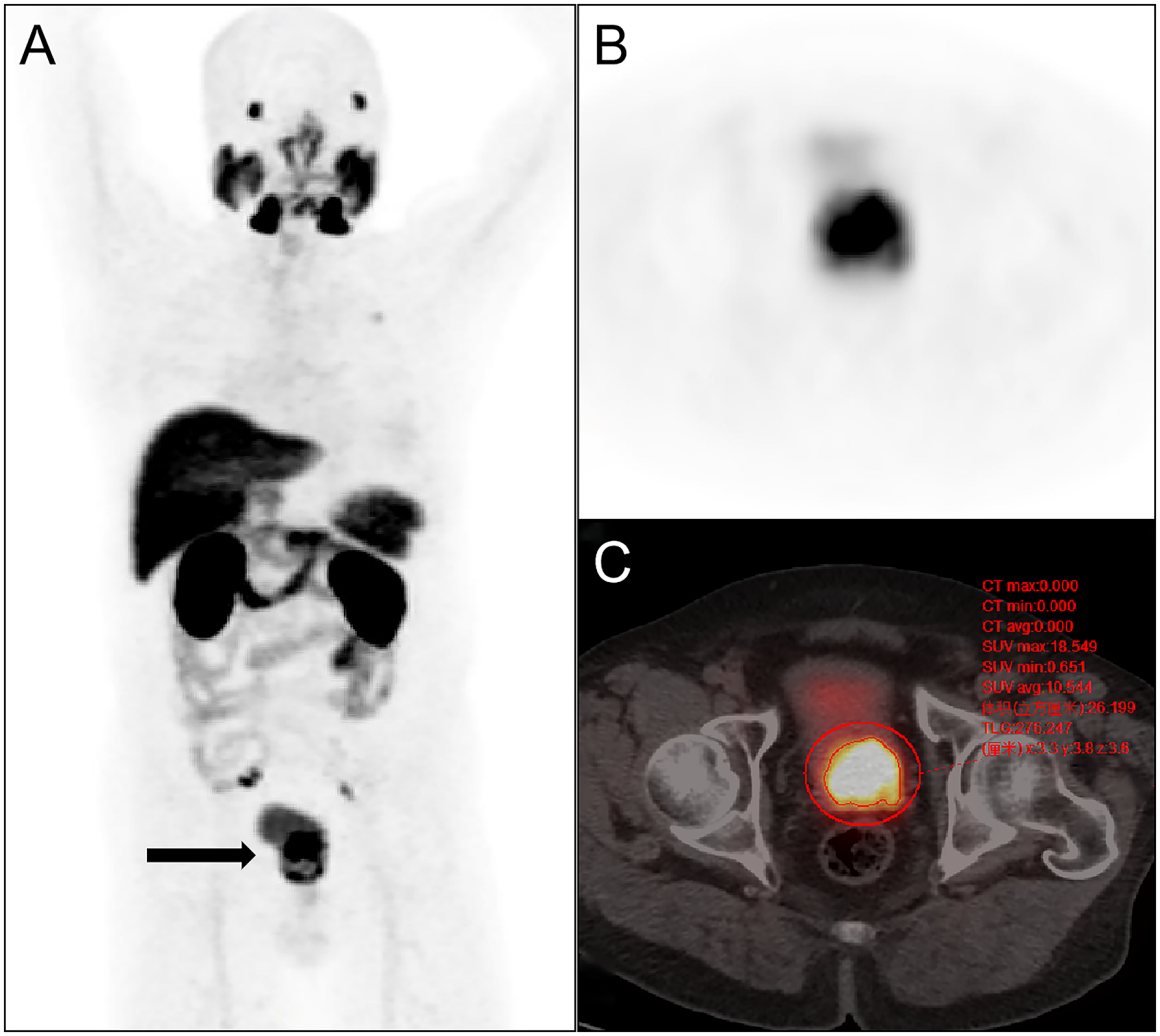
Semi-quantitative parameters of the primary prostate tumor were measured on 18F-PSMA-1007 PET/CT imaging by the 3D sketching method. **(A)** Primary prostate cancer was revealed on whole-body maximum intensity projection (MIP) imaging (arrow); **(B)** the prostate positive lesion was shown on axial PET imaging; **(C)** the volume of interest of the prostate lesion was obtained on axial fusion image (smaller red circle surrounding the lesion). SUVmax, TL-PSMAp, and PSMA-TVp of the lesion were obtained by the threshold method as 18.549,276.247 and 26.199 cm^3^.

A double-blind method was employed for the analysis of the ^18^F-PSMA-1007 PET/CT images by two nuclear medicine physicians (both with over ten years of experience in PET-CT imaging) without prior knowledge of other imaging and histopathological findings. Visual analysis was conducted specifically for lesions exhibiting higher local uptake in the prostate than in the surrounding prostatic tissue. The circular region of interest was delineated on the axial level. The positive lesions in the prostate were delineated by 40% SUVmax with a fixed threshold method. The SUVmax, PSMA-TVp, and TL-PSMAp in the lesions were recorded (**Figure 2**). The criteria for lymph node metastasis were as follows: the concentration of abnormal local radioactivity uptake in the^18^F-PSMA-1007 PET/CT image sites (except for the salivary gland, liver, gallbladder, prostate, kidney, small intestine, ganglion, and other sites where physiological uptake was visible) was judged as positive, but the known false positive lymph node uptake (axillary, mediastinal, and inguinal lymph nodes) was excluded, and the number of lymph node metastases, TL-PSMAp, PSMA-TVp, and SUVmax in the lesion were recorded. The criteria for bone metastases were as follows: lesions with increased local skeletal uptake of PSMA, excluding known fractures, degeneration, or other benign bone diseases. All cases were verified by surgical resection, histopathological biopsy results, or clinical follow-up data, while lesions that were difficult to obtain histopathologically (such as bone metastasis and distant organ metastasis) were verified by simultaneous imaging and comprehensive clinical follow-up evaluation.

### Statistical analysis

A double-blind method was employed for the analysis of the ^18^F-PSMA-1007 PET/CT images by the physicians. The data were analyzed using SPSS, and the predictive power was assessed using the receiver operating characteristic (ROC) curve. Statistical significance was determined at a threshold of P<0.05.

The data was analyzed using SPSS (version 26.0; IBM Corp., Armonk, NY, USA). Descriptive analyses of clinical and imaging data were performed. The normally distributed data are presented as mean ± standard deviation, and quartiles are expressed for data that were not normally distributed. The TL-PSMAp, PSMA-TVp, SUVmax, TPSA, and GS were compared between the groups using multi-group one-way analysis of variance and *post-hoc* tests for PCa without metastasis, oligometastasis, and extensive metastasis. The predictive power was assessed using the ROC curve. MedCalc was used to compare the differences between the area under the ROC curves(AUCs) of TPSA, GS, SUVmax, TL-PSMAp, and PSMA-TVp. Statistical significance was determined at a threshold of P<0.05.

## Results

### General data of patients with PCa

**Table 1** shows the general data of patients with PCa. Overall, 110 patients with a median age of 70 (range, 53–87) years were included. Among them, 108 patients had acinar carcinoma of the prostate, 1 had signet ring cell carcinoma of the prostate, and 1 had intraductal carcinoma. The median TPSA was 33.98 (5.42–710.00) ng/mL, which was >20 ng/mL in 60% (66/110) of the cases. GS ranged between 6–10 and it was>8 in 60.9%(67/110) of the cases.

**Table 1:** General data of the participants.

Characteristic	Numerical value
Age (years)	69.4 ± 7.3
TPSA (ng/mL)	58.95 ± 89.52
TPSA ≤ 20 ng/mL	44 (40.0%)
TPSA>20 ng/mL	66 (60.0%)
GS Score (%)	
6	18 (16.4%)
7	25 (22.7%)
8	24 (21.8%)
9	27 (24.5%)
10	16 (14.5%)
Number of transfers (%)	
No metastasis	37 (33.6%)
Oligometastasis	29 (26.4%)
Extensive metastasis	44 (40.0%)
Metastatic site	
Localized intraregional lymph node metastasis	38 (34.5%)
Extra-regional lymph node metastasis	21(19.1%)
Bone metastasis	54 (49.1%)
Visceral metastasis (lung)	7 (6.4%)

PCa did not metastasize in 37 patients and metastasized in 73; oligometastasis occurred in 29 patients; extensive metastasis in 44; lymph node metastasis in 59; bone metastasis in 54 and visceral metastasis(lung) occurred in 7 patients.

### PSMA PET/CT volumetric parameters, TPSA, and GS in the diagnosis of PCa metastasis

**Figure 3:**
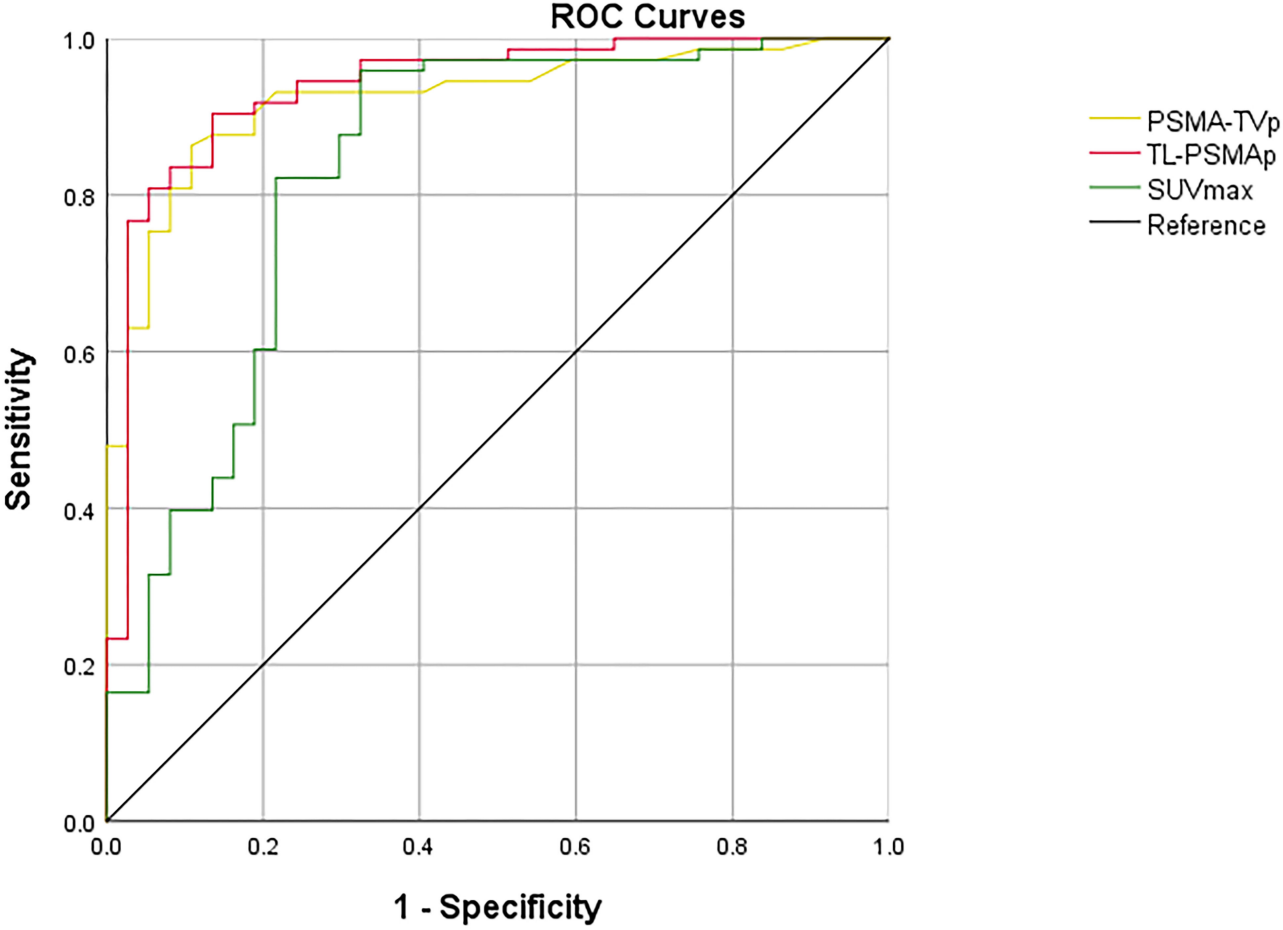
Comparison of ROC curves of SUVmax, TL-PSMAp, and PSMA-TVp in diagnosis of PCa metastasis.

**Figure 4:**
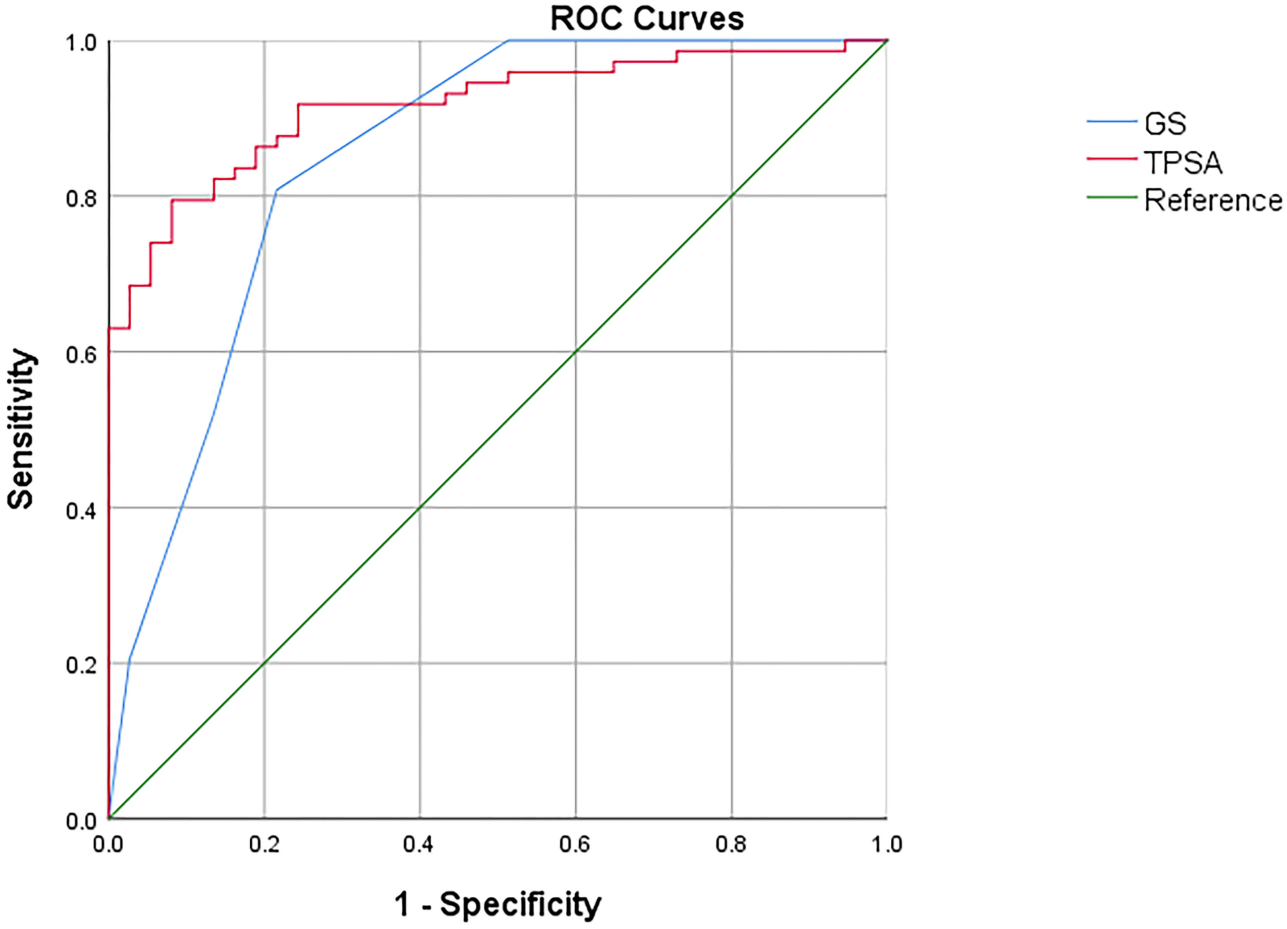
Comparison of ROC curves of GS, TPSA in diagnosis of PCa metastasis.

ROC curves were employed to assess the efficacy of GS, TPSA, SUVmax, TL-PSMAp, and PSMA-TVp in the detection of metastasis in PCa, with the highest AUCs shown for TL-PSMAp and PSMA-TVp (0.938 and 0.923, respectively; **Figure 3**). The AUC of GS, TPSA, and SUVmax was 0.851,0.916, and 0.834, respectively; (**Figure 4**). The best diagnostic thresholds were GS>7.5, TPSA>28.445 ng/mL, SUVmax>12.74, TL-PSMAp>43.162, and PSMA-TVp>7.677. The corresponding sensitivities and specificities at these thresholds were 80.8 and 78.4%, 79.5 and 91.9%, 88.3 and 86.7%, 90.4 and 86.5%, and 81.0 and 93.3%, respectively.

We further compared the GS, TPSA, SUVmax, TL-PSMAp, and PSMA-TVp AUCs for predicting PCa metastasis using MedCalc. The results showed that only the AUCs for TL-PSMAp and SUVmax, were significantly different, with Z values of 2.520 (95% confidence interval [CI]: 0.0234–0.187), P values were 0.012 in both.

### Univariate comparison of PET/CT volumetric parameters clinical and pathological characteristics for the identification of oligometastases in PCa

Statistically significant between-group differences were found in GS, TPSA, SUVmax, TL-PSMAp, and PSMA-TVp without metastases, with oligometastases, and with extensive metastases (**Table 2**). GS, TPSA, SUVmax, TL-PSMAp, and PSMA-TVp showed an upward trend with an increasing number of metastases. *Post-hoc* tests revealed that the differences in GS, TPSA, SUVmax, TL-PSMAp, and PSMA-TVp between the non-metastatic and oligometastatic groups were significant; all P values were ≤0.000. The differences in GS, TPSA, SUVmax, TL-PSMAp, and PSMA-TVp between the non-metastatic and extensive metastatic groups were significant (P=0.000), and the differences in TL-PSMAp and PSMA-TVp between oligometastatic and extensive metastatic groups were also significant (P=0.039 and 0.015, respectively, **Figure 4**) while those among GS, TPSA, and SUVmax were not significant (P=0.364, 0.900, and 0.375, respectively). TL-PSMAp and PSMA-TVp could distinguish between oligometastatic and extensive metastases; however, neither GS, TPSA, nor SUVmax could distinguish between oligometastatic and extensive metastases.

**Table 2:** Comparison of GS, TPSA, SUVmax, TL-PSMAp, and PSMA-TVp between patients with PCa with different metastatic states.

	Without metastasis	Oligometastasis	Extensive metastasis	F value	P value
GS	6.89 ± 1.13	8.31 ± 1.07	8.68 ± 0.983	30.778	0.000
TPSA	14.121 ± 8.81	72.59 ± 97.75	87.68 ± 106.45	8.197	0.000
SUVmax	10.71 ± 7.22	19.99 ± 11.53	24.65 ± 15.39	13.458	0.000
TL-PSMAp	30.79 ± 49.76	160.46 ± 135.90	336.19 ± 423.37	12.244	0.000
PSMA-TVp	3.97 ± 3.82	16.88 ± 13.07	28.74 ± 21.78	25.691	0.000

### Evaluation of patient follow-up screening

In order to verify the role of SUVmax, TL-PSMAp, and PSMA-TVp PET/CT in the evaluation of patient prognosis, 83 patients were followed up for 25 months (range, 8-48 months), and lost follow-up of 27 patients (**Table 3**). Within this group, 22 patients were identified as having no metastasis, 22 had oligometastasis, and 39 had extensive metastasis. Among the patients without metastasis, 20 patients underwent radical prostatectomy and 2 patients underwent androgen deprivation therapy (ADT) treatment. Notably, none of these patients exhibited signs of progressive disease. Out of the patients with oligometastasis, 9 patients underwent radical prostatectomy after ADT treatment, and no significant progress was observed during follow-up. Median progression-free survival (PFS) was 23 months, 13 were treated with ADT, and 2 patients were considered as progressive disease, with a mean PFS 20months. 39 patients with multiple metastases were treated with ADT. During the follow-up period, 2 patients died, and Median PFS was 14 months.

**Table 3:** Follow-up of PCa patients with different metastatic states after treatment.

	Treatment	SUVmax	PSMA-TVp	TL-PSMAp	Progression-Free Survival(months)
Without metastasis	ADT or radical prostatectomy	10.05	3.28	24.30	23
Oligometastasis	ADT+radical prostatectomy	25.76	14.40	192.81	23
	ADT alone	17.62	15.62	168.28	20
Extensive metastasis	ADT alone	24.17	26.87	282.46	14

## Discussion

Oligometastasis in PCa is an intermediate state between local tumors and extensive metastasis. Directed therapy for oligometastases has been demonstrated in several prospective studies to significantly improve survival outcomes and delay systemic therapy (7, 8). Hence, it is imperative to precisely ascertain the oligometastatic state in PCa.

Many studies have shown that PSMA PET/CT can identify metastases more effectively than conventional imaging and is more promising for the staging of patients with high-risk PCa (28–30). However, SUVmax, which is a frequently utilized parameter in PET/CT for identifying oligometastatic PCa, has been investigated in limited instances (26, 31, 32). Furthermore, a single semi-quantitative index of SUVmax lacks an accurate assessment of the overall tumor burden. Therefore, in this work, we evaluated the effectiveness and feasibility of ^18^F- PSMA-1007 PET/CT volumetric parameters for predicting oligometastatic PCa.

### Predictive capability of volumetric parameters ^18^F-PSMA-1007 PET/CT for PCa metastasis

The volumetric parameter is a useful indicator of tumor burden and invasiveness and can provide a more comprehensive reflection of the overall tumor metabolism in comparison to the SUVmax. Schmuck et al. studied the clinical significance of TL-PSMA and PSMA-TV in biochemical recurrence PCa patients for the first-time (33). The results demonstrated that the volumetric parameters TL-PSMA and PSMA-TV could more accurately assess the tumor burden of recurrent and metastatic lesions than the conventional metabolic parameter SUVmax. Liu et al. studied the volumetric parameters of ^68^Ga-PSMA- 617 PET/CT and showed that the model with TL-PSMA and PSMA-TV as core data could predict the risk of PCa transfer with the AUCs of 0.863 and 0.848, respectively, whereas the SUVmax model failed to predict the risk (34).

Karyagar et al. conducted a study to examine the variations in SUVmax, SUVmean, PSMA-TV, and TL-PSMA in patients diagnosed with primary PCa without metastasis, localized metastasis, and distant metastasis (35). The findings indicated that SUVmax and SUVmean did not exhibit any significant differences among the three groups. However, significant distinctions were observed between PSMA-TV and TL-PSMA in the non-metastasis group, the limited metastasis group, and the distant metastasis group. Notably, no statistically significant difference was found between the limited metastasis group and the distant metastasis group. It has been suggested that SUVmax and SUVmean cannot distinguish PCa from metastasis. TL-PSMA and PSMA-TV were useful to distinguish the presence or absence of metastases, but not lymph node metastasis from bone or visceral metastasis. Finally, the number of metastatic lesions was not assessed. Wang et al (26). studied the value of ^18^F-PSMA-1007 PET/CT SUVmax, GS, and TPSA in differentiating oligometastatic PCa. The results showed that the SUVmax, TPSA, and GS in the extensive metastatic group were higher than those in the non-metastatic and oligometastatic groups (all P<0.05); however, there was no difference between the non-metastatic and oligometastatic groups. SUVmax (90.50%) was more sensitive than TPSA (57.14%) or GS (55.61%). However, Wang et al (26). did not investigate the PSMA-TV of volumetric parameters or the clinical value of TL-PSMA in predicting distant metastasis. It remains unknown whether the volumetric parameters, PSMA-TV and TL-PSMA, are superior to SUVmax.

Our results showed that GS, TPSA, SUVmax, TL-PSMAp, and PSMA-TVp were higher in the metastatic group than in the non-metastatic group. GS, TPSA, SUVmax, TL-PSMAp, and PSMA-TVp may be important indicators for risk stratification in PCa metastasis and could effectively distinguish metastasis. TL-PSMAp > 43.162 and PSMA-TVp > 7.677 are at a higher risk of developing metastasis, indicating a need for more aggressive treatment. Karyagar et al (35). have demonstrated that TL-PSMAp and PSMA-TVp, as primary prostate lesion markers, are robust indicators for predicting metastasis in prostate cancer patients with a Gleason score exceeding 7.

While GS score, TPSA, SUVmax, TL-PSMAp, and PSMA-TVp have proven to be useful in discerning the presence or absence of metastasis, there are few reports on effective differentiation of oligometastasis.

In our study, based on the number of lesions, the metastatic group was divided into oligometastasis and extensive metastasis groups. The results showed that TL-PSMAp and PSMA-TVp could distinguish between oligometastases and extensive metastases. However, GS, TPSA, and SUVmax could not distinguish oligometastasis from extensive metastasis, suggesting that TL-PSMAp and PSMA-TVp have the potential to reflect the tumor burden, whereas GS, TPSA, and SUVmax do not.

In order to verify the role of SUVmax, TL-PSMAp, and PSMA-TVp PET/CT in the evaluation of patient prognosis, we followed up patients after treatment. In the follow-up period, the disease did not progress in the non-metastatic group. In the oligometastatic group, the ADT combined radical prostatectomy group had higher SUVmax, TL-PSMAp, and PSMA-TVp, indicating a higher tumor burden, However, PFS was longer than in the ADT group alone. Patients with extensive metastasis had the shortest PFS on ADT. Evidence suggests that in individuals with oligometastasis, the implementation of active treatment for both primary and metastatic lesions may result in a more favorable prognosis, our study is consistent with previous research (36, 37). Additionally, it suggests that patients with elevated SUVmax, TL-PSMAp, and PSMA-TVp values for primary lesions may have a poorer prognosis compared to those with lower values, which needs to be further verified by large-scale prospective studies. Therefore, the volumetric parameters of the noninvasive whole-body imaging method, ^18^F-PSMA-1007 PET/CT, can accurately reflect the degree of malignancy, stage, tumor burden, and invasiveness of the tumor and has a certain potential for evaluating the tumor burden of PCa.

This study circumvented the shortcomings of previous studies to some extent, analyzed the differences in SUVmax, TL-PSMAp, and PSMA-TVp of PET/CT in patients with different numbers of metastatic lesions (none, oligometastatic, and extensive metastasis), and selected the most clinically significant metabolic parameters of PET/CT that truly reflected tumor behavior, distribution, and invasiveness.

The main limitation of this study was its retrospective, single-center, small-sample design. We only selected patients with newly diagnosed PCa and did not include those with oligo-recurrence or oligometastasis after treatment. This aspect needs to be expanded upon in future studies, and a prospective study design is required to improve the reliability of the results. In addition, a biopsy of each metastatic lesion to verify positive lesions on ^18^F-PSMA-1007 PET/CT is impractical. The PSMA-TVp and TL-PSMAp values calculated using the threshold method in this study inevitably have systematic errors, and other thresholds have not been explored.

In summary, TL-PSMAp, and PSMA-TVp of ^18^F-PSMA-1007 PET/CT volumetric parameters in patients with newly diagnosed PCa differed among patients with different numbers of metastatic lesions (no metastasis, oligometastatic, and extensive metastasis) and were more advantageous than SUVmax, Therefore, ^18^F-PSMA-1007 PET/CT should be considered in patients with newly diagnosed PCa before developing a treatment plan, and more attention should be paid to TL-PSMAp of metabolic volumetric parameters.

## Data availability statement

The original contributions presented in the study are included in the article/supplementary material. Further inquiries can be directed to the corresponding authors.

## Ethics statement

The studies involving humans were approved by Ningxia Medical University Research Ethics Review Committee. The studies were conducted in accordance with the local legislation and institutional requirements. The participants provided their written informed consent to participate in this study. Written informed consent was obtained from the individual(s) for the publication of any potentially identifiable images or data included in this article.

## Author contributions

YL: Writing – original draft, Writing – review & editing, Data curation. JC: Writing – original draft. XW: Investigation, Writing – original draft. JY: Methodology, Writing – original draft. PY: Writing – original draft, Software. QZ: Writing – review & editing. JL: Supervision, Writing – review & editing.

## References

[1] Pond GR Sonpavde G de Wit R Eisenberger MA Tannock IF Armstrong AJ. The prognostic importance of metastatic site in men with metastatic castration-resistant prostate cancer. Eur Urol (2014) 65:3–6. doi: 10.1016/j.eururo.2013.09.024 24120464

[2] Zhao F Wang J Chen M Chen D Ye S Li X. Sites of synchronous distant metastases and prognosis in prostate cancer patients with bone metastases at initial diagnosis: a population-based study of 16,643 patients. Clin Transl Med (2019) 8:30. doi: 10.1186/s40169-019-0247-4 31784868 PMC6884608

[3] Peng S Chen X Huang C Yang C Situ M Zhou Q. UBE2S as a novel ubiquitinated regulator of p16 and β-catenin to promote bone metastasis of prostate cancer. Int J Biol Sci (2022) 18:3528–43. doi: 10.7150/ijbs.72629 PMC9134922 35637955

[4] Akaza H Onozawa M Hinotsu S. Prostate cancer trends in Asia. World J Urol (2017) 35:859–65. doi: 10.1007/s00345-016-1939-7 27644231

[5] Liu X Yu C Bi Y Zhang ZJ. Trends and age-period-cohort effect on incidence and mortality of prostate cancer from 1990 to 2017 in China. Public Health (2019) 172:70–80. doi: 10.1016/j.puhe.2019.04.016 31220754

[6] Haffner MC Zwart W Roudier MP True LD Nelson WG Epstein JI. Genomic and phenotypic heterogeneity in prostate cancer. Nat Rev Urol (2021) 18:79–92. doi: 10.1038/s41585-020-00400-w 33328650 PMC7969494

[7] Jadvar H Abreu AL Ballas LK Quinn DI. Oligometastatic prostate cancer: current status and future challenges. J Nucl Med (2022) 63:1628–35. doi: 10.2967/jnumed.121.263124 PMC9635685 36319116

[8] Mercier C Dirix P Meijnders P Vermeulen P Van Laere S Debois H. A phase I dose-escalation trial of stereotactic ablative body radiotherapy for non-spine bone and lymph node metastases (DESTROY-trial). Radiat Oncol (2018) 13:152. doi: 10.1186/s13014-018-1096-9 30126440 PMC6102883

[9] Rii J Sakamoto S Yamada Y Takeshita N Yamamoto S Sazuka T. Prognostic factors influencing overall survival in *de novo* oligometastatic prostate cancer patients. Prostate (2020) 80:850–8. doi: 10.1002/pros.24016 32501559

[10] Lanfranchi F Belgioia L Marcenaro M Zanardi E Timon G Riondato M. Oligometastatic prostate cancer treated with metastasis-directed therapy guided by positron emission tomography: does the tracer matter? Cancers (Basel) (2023) 15:323–30. doi: 10.3390/cancers15010323 PMC9818332 36612319

[11] Bae SH Jang WI Kang HC Kim YI Kim YH Kim WC. Current usage of stereotactic body radiotherapy for oligometastatic prostate cancer in Korea: patterns of care survey (KROG 19-08). Ann Transl Med (2021) 9:1291. doi: 10.21037/atm-21-1116 34532428 PMC8422114

[12] Palacios-Eito A Béjar-Luque A Rodríguez-Liñán M García-Cabezas S. Oligometastases in prostate cancer: Ablative treatment. World J Clin Oncol (2019) 10:38–51. doi: 10.5306/wjco.v10.i2.38 30815370 PMC6390116

[13] Lehrer EJ Singh R Wang M Chinchilli VM Trifiletti DM Ost P. Safety and survival rates associated with ablative stereotactic radiotherapy for patients with oligometastatic cancer: A systematic review and meta-analysis. JAMA Oncol (2021) 7:92–106. doi: 10.1001/jamaoncol.2020.6146 33237270 PMC7689573

[14] Awenat S Piccardo A Carvoeiras P Signore G Giovanella L Prior JO. Diagnostic role of (18)F-PSMA-1007 PET/CT in prostate cancer staging: A systematic review. Diagnostics (Basel) (2021) 11:552. doi: 10.3390/diagnostics11030552 33808825 PMC8003688

[15] Alongi F Fersino S Giaj Levra N Mazzola R Ricchetti F Fiorentino A. Impact of 18F-choline PET/CT in the decision-making strategy of treatment volumes in definitive prostate cancer volumetric modulated radiation therapy. Clin Nucl Med (2015) 40:e496–500. doi: 10.1097/RLU.0000000000000841 26053712

[16] Schuster DM Nanni C Fanti S. PET tracers beyond FDG in prostate cancer. Semin Nucl Med (2016) 46:507–21. doi: 10.1053/j.semnuclmed.2016.07.005 PMC5117950 27825431

[17] Evangelista L Briganti A Fanti S Joniau S Reske S Schiavina R. New clinical indications for (18)F/(11)C-choline, new tracers for positron emission tomography and a promising hybrid device for prostate cancer staging: a systematic review of the literature. Eur Urol (2016) 70:161–75. doi: 10.1016/j.eururo.2016.01.029 26850970

[18] Joice GA Rowe SP Pienta KJ Gorin MA. Oligometastatic prostate cancer: shaping the definition with molecular imaging and an improved understanding of tumor biology. Curr Opin Urol (2017) 27:533–41. doi: 10.1097/MOU.0000000000000449 28863016

[19] Pomykala KL Czernin J Grogan TR Armstrong WR Williams J Calais J. Total-body (68)Ga-PSMA-11 PET/CT for bone metastasis detection in prostate cancer patients: potential impact on bone scan guidelines. J Nucl Med (2020) 61:405–11. doi: 10.2967/jnumed.119.230318 PMC7067527 31541035

[20] Hawkey NM Sartor AO Morris MJ Armstrong AJ. Prostate-specific membrane antigen-targeted theranostics: past, present, and future approaches. Clin Adv Hematol Oncol (2022) 20:227–38. PMC9423035 35389387

[21] Vlachostergios PJ Zachos I Tzortzis V. Biomarkers in prostate-specific membrane antigen theranostics. Diagnostics (Basel) (2021) 11:1108. doi: 10.3390/diagnostics11061108 34207069 PMC8235046

[22] Patel NA Reiter RE. Impact of a novel molecular imaging modality, prostate-specific membrane antigen positron emission tomography, on the management of prostate cancer. J Clin Oncol (2022) 40:1497–9. doi: 10.1200/JCO.21.02940 PMC9061148 35201894

[23] Carlucci G Ippisch R Slavik R Mishoe A Blecha J Zhu S. (68)Ga-PSMA-11 NDA approval: A novel and successful academic partnership. J Nucl Med (2021) 62:149–55. doi: 10.2967/jnumed.120.260455 PMC8679592 33443068

[24] Yuk HD Byun SS Hong SK Lee H. The tumor volume after radical prostatectomy and its clinical impact on the prognosis of patients with localized prostate cancer. Sci Rep (2022) 12:6003. doi: 10.1038/s41598-022-09431-2 35397645 PMC8994775

[25] Dong S Li Y Chen J Li Y Yang P Li J. (18)F-PSMA-1007 PET/CT-derived semi-quantitative parameters for risk stratification of newly diagnosed prostate cancer. Front Oncol (2022) 12:1025930. doi: 10.3389/fonc.2022.1025930 36568229 PMC9768475

[26] Wang Z Zheng A Li Y Dong W Liu X Yuan W. (18)F-PSMA-1007 PET/CT performance on risk stratification discrimination and distant metastases prediction in newly diagnosed prostate cancer. Front Oncol (2021) 11:759053. doi: 10.3389/fonc.2021.759053 34778079 PMC8581554

[27] Cardinale J Schäfer M Benešová M Bauder-Wüst U Leotta K Eder M. Preclinical evaluation of (18)F-PSMA-1007, a new prostate-specific membrane antigen ligand for prostate cancer imaging. J Nucl Med (2017) 58:425–31. doi: 10.2967/jnumed.116.181768 27789722

[28] Kesch C Kratochwil C Mier W Kopka K Giesel FL. (68)Ga or (18)F for prostate cancer imaging. J Nucl Med (2017) 58:687–8. doi: 10.2967/jnumed.117.190157 28408526

[29] Evangelista L Maurer T van der Poel H Alongi F Kunikowska J Laudicella R. [(68)Ga]Ga-PSMA versus [(18)F]PSMA positron emission tomography/computed tomography in the staging of primary and recurrent prostate cancer. A Systematic Rev Literature. Eur Urol Oncol (2022) 5:273–82. doi: 10.1016/j.euo.2022.03.004 35367165

[30] Arnfield EG Thomas PA Roberts MJ Pelecanos AM Ramsay SC Lin CY. Clinical insignificance of [(18)F]PSMA-1007 avid non-specific bone lesions: a retrospective evaluation. Eur J Nucl Med Mol Imaging (2021) 48:4495–507. doi: 10.1007/s00259-021-05456-3 34136957

[31] Bagguley D Ong S Buteau JP Koschel S Dhiantravan N Hofman MS. Role of PSMA PET/CT imaging in the diagnosis, staging and restaging of prostate cancer. Future Oncol (2021) 17:2225–41. doi: 10.2217/fon-2020-1293 33724868

[32] Pianou NK Stavrou PZ Vlontzou E Rondogianni P Exarhos DN Datseris IE. More advantages in detecting bone and soft tissue metastases from prostate cancer using (18)F-PSMA PET/CT. Hell J Nucl Med (2019) 22:6–9. doi: 10.1967/s002449910952 30843003

[33] Schmuck S von Klot CA Henkenberens C Sohns JM Christiansen H Wester HJ. Initial experience with volumetric (68)Ga-PSMA I&T PET/CT for assessment of whole-body tumor burden as a quantitative imaging biomarker in patients with prostate cancer. J Nucl Med (2017) 58:1962–8. doi: 10.2967/jnumed.117.193581 28522740

[34] Liu C Liu T Zhang N Liu Y Li N Du P. (68)Ga-PSMA-617 PET/CT: a promising new technique for predicting risk stratification and metastatic risk of prostate cancer patients. Eur J Nucl Med Mol Imaging (2018) 45:1852–61. doi: 10.1007/s00259-018-4037-9 29717333

[35] Karyagar SS Karyagar S Guven O. Correlations of the (68)Ga-PSMA PET/CT derived primary prostate tumor PSMA expression parameters and metastatic patterns in patients with Gleason Score >7 prostate cancer. Hell J Nucl Med (2020) 23:120–4. doi: 10.1967/s002449912100 32716402

[36] Koo KC Dasgupta P. Treatment of oligometastatic hormone-sensitive prostate cancer: A comprehensive review. Yonsei Med J (2018) 59:567–79. doi: 10.3349/ymj.2018.59.5.567 PMC5990677 29869454

[37] Sabbagh A Mohamad O Lichter KE Hope TA. Management of patients with recurrent and metachronous oligometastatic prostate cancer in the era of PSMA PET. Cancers (Basel) (2022) 14:6194. doi: 10.3390/cancers14246194 36551678 PMC9777467

